# Assessing measurement invariance of familism and parental respect across race/ethnicity in adolescents

**DOI:** 10.1186/1471-2288-12-61

**Published:** 2012-04-30

**Authors:** Jeremy NV Miles, Regina A Shih, Joan S Tucker, Annie Zhou, Elizabeth J D’Amico

**Affiliations:** 1 RAND Corporation, 1776 Main St, PO Box 2138, Santa Monica, CA, 90407-2138, USA

## Abstract

**Background:**

Familism and parental respect are culturally derived constructs rooted in Hispanic and Asian cultures, respectively. Measures of these constructs have been utilized in research and found to predict delays in substance use initiation and reduced levels of use. However, given that these measures are explicitly designed to tap constructs that are considered important by different racial/ethnic groups, there is a risk that the measurement properties may not be equivalent across groups.

**Methods:**

This study evaluated the measurement equivalence of measures of familism and parental respect in a large and diverse sample of middle school students in Southern California (n = 5646) using a multiple group confirmatory factor analysis approach.

**Results:**

Results showed little evidence of measurement variance across four racial/ethnic groups (African American, Hispanic, Asian, and non-Hispanic White), supporting the continued use of these measures in diverse populations. Some differences between latent variable means were identified – specifically that the Hispanic group and the white group differed on familism.

**Conclusions:**

No evidence of invariance was found. However, the item distributions were highly positively skewed, indicating a tendency for youth to endorse the most positive response, which may reduce the reliability of the measures and suggests that refinement is possible.

## Background

### Cultural values

When individuals move from the country in which they were born, they continue to hold values and practice beliefs that are considered important in their home culture. The values held by the group may be passed on to their children and thereby retained by the cultural group [1,2]. For example, familism is a belief system in which the needs of the wider family are thought to override the needs of the individual within that family, and is often described as a value rooted in Hispanic culture. In addition, parental respect (also known as filial piety) is a cultural value emphasized in many Asian cultures [2-4]. Studies have found that adolescents who more strongly endorse the values of familism and parental respect tend to engage in less use of alcohol and other drugs [5-7]. Parental respect has also been found to be associated with reduced heavy drinking [8], misconduct [9] and smoking [7], and increased life satisfaction [10] and desire for academic achievement [11]. Research that has focused on Hispanic adolescents has found that those who report higher familism have decreased risk for cigarette initiation [3] and heavy drinking [12].

Shih and colleagues [13] recently examined the potential of these constructs to mediate racial/ethnic differences in reports of lifetime alcohol and other drug (AOD) use. A mediating variable is one which explains the effects from a predictor variable to an outcome variable [14,15]. Cultural values have the potential to act as mediators which may partially explain the differences in the association between race/ethnicity and AOD use. [12]. Shih and colleagues [13] found that parental respect partially mediated the lower consumption among Asian youth (compared to non-Hispanic white adolescents); however familism did not mediate the higher consumption found among Hispanic youth compared to non-Hispanic white adolescents. These results suggest that cultural values may partially explain ethnic/racial differences in use of AOD, and may also provide a mechanism through which interventions may be designed to operate. However, if such research is undertaken, it is important to ascertain that the measures are appropriate for all ethnic groups.

### Potential for invariance

Given that familism and respect are based on values rooted in one particular culture, it is possible that the items that comprise these measures may not be equivalent in members of other cultural groups. Thus an issue that arises in the use of psychometric instruments in research comparing groups of individuals is that some items may be interpreted differently by different groups, or that some items might represent different constructs in different groups. When a test is found to measure a construct equivalently across groups, the test is referred to as having factorial invariance [16]. In the item response theory literature, the same phenomenon is referred to as differential item functioning (DIF; [17,18]). When differences in measurement properties exist because of lack of invariance, then measurements from those instruments will not have ‘construct comparability’ – that is, they will not be assessing the same construct in each group, and so analyses based on such tests will be possibly suspect.

Although some existing studies have provided evidence for measurement invariance across ethnic groups of cultural value measures, these studies have focused on adult-only, or Hispanic-only populations [1,2,19], which potentially limits generalizability to other populations.

### Describing and detecting invariance

Invariance can be assessed using a multiple group confirmatory factor analysis based approach such that in a series of steps, restrictions are added to the model and the decrement in fit is noted at each step. If it is determined that the model fit is substantially worse when restrictions are added in a step, the modeling process stops and further models are not fit.

Factorial invariance is divided into four levels based on these tests of model fit, representing stronger tests of invariance. The first level is *configural invariance*. If a measurement instrument has the quality of configural invariance across groups, this means that the general factor structure is maintained across the groups. A failure of configural invariance occurs when an item is interpreted in a completely different manner by two different groups. For example, the term ‘fussy’ has very different meanings in British and American English – in British English to be fussy is to be fastidious or picky whereas in American English, to be fussy is to be easily upset. Thus, an item containing this term would likely fail a test of configural invariance across cultures.

If a measure has been shown to have configural invariance, it can then be examined for *weak invariance*. Weak invariance is said to hold if the model where factor loadings are constrained to be equal for each of the groups provides the best fit to the data. If there were group differences, such that the loading for one item were lower in one group, this would mean that the item did not function as well for that group, and therefore that the variance on the construct would be lower for that group. A lower variance is associated with attenuated relationships, and may manifest as differences between groups that are artifacts of the lack of weak invariance, particularly when examining moderators or mediators [20].

*Strong invariance* is said to occur when the intercepts (or thresholds) for each item are also constrained to be equivalent among groups. For strong invariance, the intercept of the items should not vary by group; that is, two individuals from different groups, with the same score on the factors, will have the same expected scores on each of the items. Failure to satisfy strong invariance might occur if one group were more likely to endorse a particular item given the same score. For example, we might find that males were less likely to endorse an item that asked about crying than females, despite having the same level of the underlying factor. This might be a social desirability bias (males may not admit to crying) or it may be a real difference. Whatever the reason for the difference, if such an effect occurs, incorporating an item that asks about crying will likely create a bias in the scale – females with the same underlying latent variable score as males will have higher measured scores. Miles and colleagues [21] investigated whether a lack of strong invariance might be responsible for higher levels of reported posttraumatic stress disorder in Hispanic populations and found little evidence for such an effect, thereby ruling this out as an explanation for the difference.

The key goal of testing for factorial invariance is to understand the extent to which the measurement properties of a scale are consistent in different groups. The degree of consistency, or type of invariance, has practical implications for the use of a measurement instrument and will provide theoretical information about the ways in which these constructs differ across populations. For instance, it may be that the items do not form coherent scales when applied in groups for whom that construct is not held in esteem. If this were the case, we would be likely to detect a lack of configural invariance, and such a result would suggest that the use of the scales in some groups would be inappropriate. If weak invariance were not found, that is if the factor loadings *do* differ across groups, it would suggest that the reliability differs across groups. Groups for whom reliability is lower would have greater error variance and hence weaker relationships would be detected between the cultural value measure and any other measures, even when the relationship between the underlying latent variables was equal. In this situation, conclusions about group differences in these associations may be erroneous in that they may be an artifact of differences in the reliability of the cultural value measure. A failure of strong invariance would suggest that certain items in the scale were biased for some groups. Lack of strong invariance would make comparisons across groups difficult, as it suggests that some groups would have higher scores, but that this would be an artifact of the measurement properties rather than a true difference.

### Aims

Cultural values such as familism and parental respect are associated with substance use among middle school adolescents and are commonly-used scales in cross cultural research. Use of these measures makes the assumption that factorial invariance is present in the data, but this assumption is rarely tested. Although the measurement instruments have been found to be invariant across different adult racial/ethnic groups, they may not be suitable for use with diverse adolescent populations. The current study assessed the presence of measurement invariance of familism and parental respect across four racial/ethnic groups in a large sample of 10–15 year old adolescents in Southern California to inform whether meaningful comparisons could be made across racial/ethnic groups using these measures.

## Methods

### Sample

Sixteen schools across three school districts in Southern California were recruited to take part in a cluster randomized trial of an after-school prevention program called CHOICE (see [22]). The data presented in this paper were collected at baseline, prior to the implementation of the program. A total of 14,797 students across all 16 schools received parental consent forms, 92% of parents returned the forms, and 71% of those gave permission for their child to participate in the study. Surveys were completed at school on a pre-scheduled day during physical education classes in the Fall of 2008, and 91% of eligible students took part in the survey. To be included in the analytic sample, respondents needed to complete the survey and describe themselves as being in one of the four ethnic/racial groups non-Hispanic black, Asian, Hispanic or non-Hispanic white. The scales analyzed in this paper were included at the end of the questionnaire, and not all students reached them due to time constraints.

The analytic sample comprised 5646 students. 23% of the sample was Asian (n = 1306), 4% of the sample was non-Hispanic black (n = 230; referred to hereon as black), 52% of the sample was Hispanic (n = 2947), and 21% of the sample was non-Hispanic white (n = 1163; referred to as white). Age ranged from 10 to 15 years (mean 12.2, SD 0.92) and 51% of the sample was female. Responses were protected by a Certificate of Confidentiality from the National Institutes of Health. All materials and procedures were approved by the institution’s Internal Review Board, school districts, and individual schools.

### Measures

In this study we focus on measures of familism and parental respect that were initially developed by Unger [23] for use with adolescents, and updated by Soto and colleagues [24]. For example, the original scale contained the item “When someone has problems, one can count on the help of relatives”; the revised scale contains the item “If one of my relatives needed a place to stay for a few months, my family would let them stay with us.” Both scales (see Table 1) were assessed with four items that were rated on a 4-point scale and averaged (1 = strongly agree to 4 = strongly disagree; alphas = 0.77 for familism and 0.89 for parental respect). Higher scores indicated greater familism and parental respect.

**Table 1:** Items from familism and parental respect measures, along with abbreviation for item

Familism measure:	
1. If one of my relatives needed a place to stay for a few months, my family would let them stay with us.	(stay)
2. I expect my relatives to help me when I need them.	(help me)
3. When a family makes an important decision, they should talk about it with their close relatives.	(talk)
4. If anyone in my family needed help, we would all be there to help them.	(help others)
Parental respect measure:	
1. I will take care of my parents when they are old.	(take care)
2. It is important to honor my parents.	(honor)
3. It is important to respect my parents.	(respect)
4. I want to be a good person so that people know that my parents raised me right.	(good person)

### Data analysis

The aim of the study was to test for factorial invariance and mean differences in latent variables across the two scales. The analysis followed the same procedure for each scale. Mplus v6.0 was used for all analyses [25] using weighted least squared with mean and variance adjustment (WLSMV) estimation and the data were treated as comprising ordered categorical measures. Because the data were treated as ordinal, the measured variables do not have an intercept, but rather the location of the items is represented as a series of thresholds.

We first explored the data by examining the proportion of individuals in each racial/ethnic group who gave a response to an item and estimating the correlation matrices for each scale for each ethnic group. We fitted a single factor model to each group to ensure that the measurement model fit appropriately.

We then fit model 1, which tested configural invariance, in order to determine whether the general factor structure (in terms of the number of latent variables) of the scales fit all four groups. This model was a multiple group model with all parameters free to vary between groups. In model 2, the weak factorial invariance model, we examined differences in loadings across the group racial/ethnic groups by constraining factor loadings to equality across all groups. If this model was tenable, we could conclude that the loadings did not differ between groups. For identification purposes, the mean of the latent variable in one group (White) is constrained to be equal to zero, the loading for item 1 in all groups is constrained to be equal to 1 (this identifies the variance of the latent variable), and the first threshold of the first item for all groups is constrained to be equal to the equivalent threshold in the White group (this identifies the means of the latent variables in all groups except the White group). Model 3 constrained measured variable thresholds to equality across groups to create the strong factorial invariance model. This model tests whether there is a difference in the probability of a response category across racial/ethnic groups, given the same score on the latent variable (meaning that an increase in score on the latent variable, as assessed with any three items, leads to a similar increase in score on the fourth item, for all four groups). Finally, we constrained the means of the latent variable in each group to equality to test for differences between groups in the latent variable score. Note that testing of strict invariance (equality of loadings, thresholds and error variances) is not possible with ordered categorical data, as the error variances are not identified independently from the thresholds.

The white group was used as the reference category. The variance of the latent variable for each group was identified by constraining the loading of item 1 to be equal across groups. The mean of the latent variables for the non-reference category was identified by constraining the first threshold on the first item to be equal to the reference category threshold for models 1 and 2. We evaluated model fit through the use of the chi-square test, comparative fit index (CFI), non-normed fit index (NNFI) and root mean square error of approximation (RMSEA). At each stage, the decrement in model fit was assessed by chi-square obtained with the Mplus *difftest* function for WLSMV models; in addition, we evaluated model fit through the use of the RMSEA and CFI, for determining invariance we followed the recommendation [26] of using a change in CFI of −0.01 to indicate a substantial change in model fit when constraining parameters across groups.

## Results

Table 2 shows the correlation matrix and endorsement probabilities for both of the scales, for each ethnic/racial group. The correlation matrices show that all items are highly correlated within scales for all groups, although for the familism scale, the correlations among items in the Asian group appear to be lower (range = 0.45 to 0.63) compared to other groups (no correlation drops below 0.60).

**Table 2:** Polychoric correlations and endorsement proportions split by ethnic/racial group

**Familism**														
	Correlation Matrix							Endorsement Proportions						
	1. stay			2. help me		3. talk about decision		Strongly Disagree		Disagree		Agree		Strongly Agree
Asian														
1. stay	1.00							0.02		0.03		0.19		0.76
2. help me	0.48			1.00				0.03		0.05		0.21		0.71
3. talk about decision	0.45			0.61		1.00		0.03		0.08		0.26		0.63
4. help others	0.52			0.58		0.63		0.02		0.04		0.16		0.78
African American														
1. stay for a while	1.00							0.06		0.06		0.17		0.72
2. help me	0.76			1.00				0.05		0.05		0.15		0.75
3. talk about decision	0.70			0.74		1.00		0.09		0.10		0.24		0.57
4. help others	0.74			0.80		0.74		0.05		0.05		0.20		0.71
Hispanic														
1. stay for a while	1.00							0.06		0.03		0.21		0.69
2. help me	0.69			1.00				0.05		0.04		0.18		0.73
3. talk about decision	0.60			0.66		1.00		0.08		0.08		0.22		0.62
4. help others	0.74			0.76		0.72		0.05		0.04		0.17		0.74
White														
1. stay for a while	1.00							0.02		0.03		0.22		0.73
2. help me	0.69			1.00				0.02		0.02		0.18		0.78
3. talk about decision	0.60			0.66		1.00		0.03		0.10		0.28		0.59
4. help others	0.74			0.76		0.72		0.02		0.04		0.16		0.79
**Parental Respect**														
		Correlation Matrix					Endorsement Proportions							
		1 Take care	2 Honor		3 Respect		Strongly Disagree		Disagree		Agree		Strongly Agree	
Asian														
1. take care		1.00					0.01		0.01		0.09		0.89	
2. honor		0.82	1.00				0.01		0.02		0.09		0.88	
3. respect		0.80	0.93		1.00		0.01		0.01		0.07		0.92	
4. good person		0.78	0.85		0.87		0.01		0.01		0.06		0.92	
African American														
1. take care		1.00					0.04		0.04		0.13		0.80	
2. honor		0.82	1.00				0.04		0.04		0.08		0.84	
3. respect		0.89	0.91		1.00		0.04		0.03		0.06		0.87	
4. good person		0.92	0.89		0.94		0.04		0.02		0.05		0.90	
Hispanic														
1. take care		1.00					0.04		0.02		0.09		0.85	
2. honor		0.86	1.00				0.04		0.02		0.11		0.83	
3. respect		0.87	0.93		1.00		0.04		0.02		0.07		0.88	
4. good person		0.85	0.91		0.92		0.04		0.01		0.08		0.87	
White														
1. take care		1.00					0.02		0.03		0.14		0.82	
2. honor		0.78	1.00				0.02		0.03		0.14		0.81	
3. respect		0.77	0.93		1.00		0.02		0.02		0.08		0.88	
4. good person		0.73	0.84		0.86		0.02		0.02		0.09		0.88	

The endorsement proportions show that for both scales, ‘strongly agree’ was the most commonly selected response, with a low of 57% of the African American sample endorsing strong agreement of item C in the familism scale (“When a family makes a decision … they should talk about it”). Familism, which is hypothesized to be a Hispanic value, did not seem to be more highly endorsed by the Hispanic sample than by the other groups. For none of the familism items were Hispanics the most likely to select the ‘strongly agree’ option. However, parental respect, which is described as an Asian value, did seem to have higher endorsement of ‘strongly agree’ by the Asian sample compared to the other groups. Across all groups, ‘strongly agree’ was selected more often for the parental respect items compared to the familism items.

Table 3 shows fit statistics for the models in which a single factor model is estimated, with parameters freely estimated across groups for both the familism and parental respect scales. The fit statistics show a good fit to the data for all groups, with the chi-square test reaching statistical significance for only one model – the Asian group, for familism. For this group, the other measures of fit are acceptable, with CFI = 0.997 and RMSEA = 0.045. These results demonstrate configural invariance.

**Table 3:** Fit statistics from separate models for each racial/ethnic group

**Familism**				
	Asian	African American	Hispanic	White
	n = 1306	n = 230	n = 2947	n = 1163
Chi-square	7.18	0.04	8.09	0.54
df	2	2	2	2
(p)	(0.028)	(0.982)	0.018	0.763
RMSEA	0.045	0.000	0.032	1.00
CFI	0.997	1.000	0.999	1.000
**Parental Respect**				
Chi-square	3.8	3.8	0.37	2.29
df	2	2	2	2
(p)	0.152	0.152	0.832	0.318
RMSEA	0.026	0.063	0.000	0.011
CFI	1.000	0.999	1.000	1.000

Table 4 shows the factor loadings and factor variances from the configural invariance model. Unstandardized factor loadings appear to be high across all items and groups. The magnitude of the factor variance for familism appears to be lower for the Asian and white groups than for the African American and Hispanic groups.

**Table 4:** Factor loadings and factor variance from configural invariance model. Unstandardized estimates are labeled Est, standardized estimates labeled St Est

**Familism**								
Item	Asian		African American		Hispanic		White	
	Est	St Est	Est	St Est	Est	St Est	Est	St Est
Loading 1	1.00	0.62	1.00	0.84	1.00	0.80	1.00	0.68
Loading 2	1.23	0.76	1.07	0.90	1.04	0.84	1.13	0.76
Loading 3	1.27	0.78	0.99	0.83	0.97	0.78	0.96	0.65
Loading 4	1.29	0.80	1.06	0.89	1.14	0.92	1.36	0.92
Factor Variance	0.38	1.00	0.71	1.00	0.65	1.00	0.46	1.00
**Parental Respect**								
Loading 1	1.00	0.85	1.00	0.93	1.00	0.90	1.00	0.81
Loading 2	1.13	0.96	1.00	0.92	1.07	0.96	1.18	0.96
Loading 3	1.15	0.97	1.05	0.97	1.09	0.97	1.19	0.97
Loading 4	1.06	0.90	1.05	0.97	1.05	0.94	1.09	0.88
Factor Variance	0.72	1.00	0.86	1.00	0.80	1.00	0.66	1.00

All estimates are high, and are statistically significant (p < 0.001).

Table 5 shows the fit indices and change in fit indices across the four models. Model 1, representing configural invariance, places no constraints on any of the parameters across groups. This model provides a very good fit to the data, with the chi-square test being non-significant in both the familism and parental respect scales and the RMSEA (0.025 for familism and 0.016 for respect) and CFI (1.00 for both scales). In model 2, the weak invariance model, the factor loadings are constrained to be equal across groups. The model chi-square was statistically significant for both scales when this constraint was added, and the ∆chi-square was statistically significant for both groups. However, it is often suggested that chi-square is overpowered in large samples such as these, hence alternatives have been suggested. One common alternative is to look at the ∆CFI, with a recommendation that a change in CFI of less than 0.01 indicates that the restriction on the parameters has not caused a substantial worsening of the model fit [26]. The change in CFI for both scales was considerably lower than 0.01 and thus we retained these constraints.

**Table 5:** Model fit statistics for the series of progressively restrictive models

**Familism**				
	Model 1 – Configural Invariance	Model 2 – Weak Invariance	Model 3 – Strong Invariance	Model 4 – Equal Factor Means
Chi-square	14.8	74.2	135.0	129.6
df	8	17	38	41
(p)	0.063	<0.001	<0.001	<0.001
RMSEA	0.025	0.049	0.043	0.039
CFI	0.999	0.996	0.993	0.993
Δ Chi-square	-	54.1	61.7	7.4
df		9	21	2
(p)		<0.001	<0.001	0.060
Δ CFI	-	−0.003	−0.003	0.000
**Parental Respect**				
	Model 1 – Configural Invariance	Model 2 – Weak Invariance	Model 3 – Strong Invariance	Model 4 – Equal factor means
Chi-square	10.7	69.2	67.0	106.5
df	8	17	38	41
(p)	0.218	<0.001	0.003	<0.001
RMSEA	0.016	0.047	0.024	0.034
CFI	1.000	0.999	0.999	0.999
Δ Chi-square	-	50.2	(1)	21.4
df		9		3
(p)		<0.001		<0.001
Δ CFI	-	−0.001	0.000	0.000

Nested model difference test failed to converge.

In model 3, we tested for differential item functioning at the threshold level, by constraining thresholds across groups. Again, the decrement in model was statistically significant, as measured by chi-square for the Familism scale (for the Parental Respect scale, the model difference test failed to converge) but the small change in CFI (0.000 and 0.003, respectively) suggested that this decrement was sufficiently small to retain this constraint on thresholds. Table 6 shows the means (with standard errors and variances) for the latent variables in each group in model 3. For both scales, the lowest mean score on the latent variable is found in the white group. In the familism scale the Asian, African American, and Hispanic groups have slightly higher means, but only the Hispanic group achieves statistical significance (p = 0.027). In the parental respect scale, the highest means are those of the African American and Hispanic groups, with both of these groups having a mean which is significantly higher than the white group (p <0.001 for Hispanic, p = 0.038 for African American). The Asian group also has a statistically significantly higher mean than the white group (p = 0.026).

**Table 6:** Latent variable means and variances (standard error) from model 3

	**Familism**		**Parental Respect**	
	Mean	Variance	Mean	Variance
Asian	0.02 (0.05)	0.49 (0.06)	0.27 (0.12)	0.27 (0.12)
African American	0.06 (0.11)	1.12 (0.25)	0.53 (0.26)	1.76 (0.58)
Hispanic	0.11 (0.05)	1.07 (0.11)	0.44 (0.11)	1.44 (0.21)
White (reference)	0.00	0.48 (0.03)	0.00	0.67 (0.03)

In model 4, the means are constrained to equality, to test for differences. In the familism scale, restricting the latent variable means to equality caused chi-square to increase a non- statistically significant degree (p = 0.060). In the parental respect scale, the increase in chi-square was larger and did achieve statistical significance; however, using the criteria of the change in CFI, the increase was not substantial. Table 7 shows the parameter estimates for the final model.

**Table 7:** Parameter estimates from model 4 – all loadings constrained to equality across groups

**Familism**	**Item 1**	**Item 2**	**Item 3**	**Item 4**
Loading	1.00	1.14	0.97	1.25
Standardized loading (1)	0.70	0.79	0.67	0.87
Thresholds (2)	−1.92	−2.09	−1.69	−2.14
	−1.60	−1.68	−1.13	−1.70
	−0.64	−0.74	−0.31	−0.82
**Parental Respect**	Item 1	Item 2	Item 3	Item 4
Loading	1.00	1.16	1.17	1.09
Standardized loading (1)	0.95	0.85	0.86	0.93
Thresholds (2)	−1.89	−1.98	−2.00	−1.94
	−1.65	−1.68	−1.77	−1.75
	−1.01	−1.01	−1.22	−1.18

(1) Standardized loadings are determined by the loading and the variance of the items and latent variable, hence whilst unstandardized loadings are constrained to equality across groups, standardized loadings will differ; standardized loadings are shown for the white group.

(2) Thresholds are the log-odds of giving a response lower than the current threshold for an individual who has a score of 0 for the latent variable.

## Discussion

This study assessed the presence of measurement invariance of familism and parental respect across four racial/ethnic groups in a sample of 10–15 year olds to inform whether meaningful results can be obtained using these measures in samples with ethnic/racial diversity. Overall, we found no evidence of substantial difference in measurement properties of the instruments across the four racial/ethnic groups, supporting the continued use of these measurement instruments in research in adolescents.

We tested whether stronger levels of invariance across four ethnic/racial groups of younger adolescents: Asians, African Americans, Hispanics, and whites would substantially worsen model fit. We found no evidence of substantial configural invariance, weak invariance or strong invariance for either scale. When we tested for strong invariance, we found small and non-significant differences. We then tested the difference between the means of the latent variables. For familism, we found that Hispanic respondents had higher means on the scale than other ethnicities, and that this group was significantly different from the white group, as may have been hypothesized, given the origins of the measure. For parental respect, the differences between means were larger, and were statistically significant; however the differences were not substantial. In addition, the highest latent variable mean scores were found in the Hispanic and African American groups, not the Asian group, which replicates previous findings in the literature [23,27].

Because of demographic differences in study populations, no studies provide directly comparable data to the present study. Villareal and colleagues [19] explored cultural comparability of a measure of familism across different Hispanic groups in the US, in both Spanish and English, in a population of adults aged 18 to 65 sampled by telephone from areas with a high population of Hispanics. They found support for invariance of their familism measure across language (Spanish versus English), and country of origin (USA, Mexico, or Latin America), suggesting that the measurement properties of familism were consistent across these groups. Schwartz [1] examined differences in the factor structure of a measure of familism across Hispanic, white and African American university students. This research also found no differences in factor structure or mean score across these groups; however the relatively low sample size of 57 white respondents and 64 African American respondents limited the power of the study to find differences that may have existed. Recently Schwartz and colleagues [2] carried out a much larger investigation of invariance for familism and parental respect in two samples. The first comprised 1000 university students and the second included over 10,000 university students (in study 2). In both samples, they compared the fit of models across four ethnic groups (Asian, African American, Hispanic, white) and found measurement equivalence across these groups. Since we found no measurement invariance in our young sample of adolescents, this suggests that similar measures of familism and parental respect scales can be applied to a diverse sample of younger populations.

Given the recent, drastic demographic shifts and historical migration patterns of racial/ethnic groups in the US, there may be racial/ethnic differences in birth country, timing of migration to the US, and acculturation among young US adolescents that do not exist in US adults [28,29]. Thus, non-equivalence of cultural values in a diverse sample of younger adolescents might be expected. If we had found differences in measurement properties across groups, the appropriate comparisons of the relationship between cultural values and substance use risk among adolescents could not be made across those groups and this would have both theoretical and practical implications. Specifically, it is theoretically important to understand differences in the structure of cultural values between racial/ethnic groups in order to understand the cognitive processes that are employed when an individual decides how to answer a particular item. It is of practical importance to ensure that there is cultural comparability of measures across groups if the measures are to be employed in diverse populations of members of many groups to ensure that the same construct is being assessed in all individuals. Our results indicate that there is no evidence of substantively important invariance, suggesting no evidence of problems with bias or differences in measurement properties when employed in a diverse sample of middle school adolescents.

The study has a number of strengths. In particular, the size of the sample was over 5000 individuals, which provided a great deal of power to estimate parameters with a high degree of precision. In addition, we tested factorial invariance by race/ethnicity among a highly diverse sample of Hispanics, Asians, African Americans, and whites. To our knowledge, this study is the first to report such a finding in middle school aged youth, hence demonstrating the appropriateness of the measures for respondents of this age group and these racial/ethnic groups. However there are limitations that should be taken into account when interpreting results. First, although the overall sample size was large, the number of African American respondents was relatively low (n = 230) compared to other groups. Second, the sample was racially/ethnically diverse, but represented youth from public schools across the Los Angeles metropolitan area, which may limit the generalizability of the results to the larger population of adolescents in the U.S; in particular Hispanic youth in the Los Angeles area are more likely to have family origins in Mexico than are youth in other areas of the US. In addition, the description of youth as ‘Asian’ may be reducing a diverse population into a single category which is useful descriptively, but includes many different cultural groups from a large and diverse geographical area and therefore may be an oversimplification. Further research might explore measurement properties of cultural value measures among sub-groups of Asians (or other groups). It is also important to note that differences may exist between first, second and third generations of immigrants – as cultural values can sometimes become diluted or incorporate values from other cultures over generations [24].

We also found that responses to individual items were highly skewed, leading to ceiling effects as a majority of youth endorsed ‘strongly agree’ for all items. The analysis approaches that we employed do not assume a normal (or any other) distribution and therefore the large number of responses at the high end of the scale reduced our ability to discriminate individuals at that level and hence may reduce power [30]. In future research, a change in the response scale or in item wording might improve the ability to discriminate at that level. Increasing the number of response categories has been shown to increase the responsiveness of a scale (e.g. [31]), however increasing the number of responses can make the responses less meaningful, particularly in younger individuals. Rephrasing the items to make them more extreme (increasing the difficulty of agreement) might also be effective, for example “I expect my relatives to help me when I need them” might become “I *definitely* expect my relatives to help me when I need them” (italics added for emphasis). However, care should be taken that such rewording does not alter the meaning of the items.

It is also possible that social desirability played a role in the ceiling effect. The confidentiality of the survey was emphasized, which should reduce socially desirable responding due to impression management. However, self-deception enhancement and denial may still have played a role [32]. Whether socially desirable responding is influencing responses on these measurement instruments is an empirical question worthy of further investigation.

## Conclusions

In sum, this study used data from a large, diverse sample of Asian, Hispanic, white and African American adolescents to rigorously examine whether specific cultural values, which have been previously linked with various adolescent health risk behaviors [6,13,33,34], can be used in racial/ethnically diverse samples of adolescents in a comparable way across groups. Our results provide the first quantitative evidence that no bias should arise if researchers would like to include these measures of cultural values in studies of young, middle school adolescents from different racial/ethnic groups to answer questions about how cultural values may influence health and well-being in early adolescence.

## Competing interests

All authors declare that they have no competing interests.

## Authors’ contributions

All authors were involved in the design of the larger study from which these data were taken, EJD is principal investigator. EJD and JST designed and oversaw the data collection exercise. JM and RS conceived and designed the study. AZ was responsible for data manipulation, checking and management, JM and AZ carried out the analysis. JM wrote the first draft of the paper, all authors then contributed. All authors have given final approval of the manuscript.

## Pre-publication history

The pre-publication history for this paper can be accessed here:

http://www.biomedcentral.com/1471-2288/12/61/prepub
